# Editorial: Exercise intervention on metabolic syndrome: focus on pyroptosis

**DOI:** 10.3389/fendo.2024.1461283

**Published:** 2024-07-29

**Authors:** Lijing Gong, Tao Tong, Enming Zhang

**Affiliations:** ^1^ Key Laboratory of Exercise and Physical Fitness of Ministry of Education, Beijing Sport University, Beijing, China; ^2^ China Institute of Sport and Health Science, Beijing Sport University, Beijing, China; ^3^ College of Food Science and Nutritional Engineering, China Agricultural University, Beijing, China; ^4^ Department of Clinical Sciences in Malmö, Lund University Diabetes Centre, Lund University, Malmö, Sweden

**Keywords:** metabolic syndrome, exercise, pyroptosis, obesity, T2DM

Metabolic syndrome (MetS) involves various dysfunctional metabolic processes, spanning from the subcellular compartments to entire organs, and is associated with serious metabolic diseases including diabetes, obesity, and cardiovascular diseases (CVDs). Exercise has been well-documented as a key interventional strategy to alleviate or treat the syndrome, though the underlying mechanisms remain elusive. For example, aerobic exercise can mitigate impaired vasodilation in diabetic mice by enhancement of BKCa channel protein expression through AMPK/Nrf2/HO-1 pathway (1). In addition, exercise can induce the release of signaling molecules (exerkines) from organs and tissues, which influence various chronic inflammation-related diseases. Thus, exercise plays an important role in regulating metabolism and augmenting the anti-inflammatory effects, providing significant advantages for MetS management (2). Recently, a cell death pattern, pyroptosis, has been shown to be inhibited in exercise-induced improvement of metabolic disorders, implying a novel target for the treatment of MetS and related diseases.

Pyroptosis is mediated by inflammasomes and a downstream effector, gasdermin D (GSDMD). The NOD-like receptor family pyrin domain-containing 3 (NLRP3) is a key cytosolic inflammasome, composed of the innate immune receptor protein NLRP3, adapter protein ASC, and inflammatory protease caspase-1. The assembled NLRP3 inflammasome can activate the protease caspase-1 to induce GSDMD-dependent pyroptosis and facilitate the release of IL-1β and IL-18, which further contribute to innate immune defense and homeostatic maintenance. However, aberrant activation of the NLRP3 inflammasome is linked with the pathogenesis of inflammatory diseases (3). For example, human and obese mice studies show that obesity and insulin resistance (IR) are associated with increased NLRP3 expression in adipose tissue (4). Accordingly, targeting the secretion of hormones (5) or activating endogenous substances (6) to resist pyroptosis could be a promising research direction for mitigating MetS.

IR and its related factors related to pyroptosis can be improved by exercise training. IR is an important pathological index in overweight/obesity and numerous metabolic disorders, prevalent even in many obese children and adolescents. It is associated with an increased risk of developing metabolic disorders in adulthood (7). Liu et al. demonstrated that activating brown adipose tissue (BAT) and browning of white adipose tissue (WAT) can protect against obesity and obesity-related metabolic disease. Approaches to activate BAT and/or browning WAT include cold exposure, exercise, hypoxia exposure, and small-molecule treatment. This study highlights the functional mechanisms of small-molecule treatment and BAT transplantation using batokine, the sympathetic nervous system, and/or the gut microbiome. Finally, they discussed the causality between body weight loss induced by bariatric surgery, exercise, and BAT activity. Moreover, Kazeminasab et al. explored the effects of exercise training on IR in children and adolescents who are overweight or obese. The reports indicate that exercise is effective for lowering fasting glucose, fasting insulin, HOMA-IR, and BW in children and adolescents who are overweight or obese, providing an important strategy for controlling IR and its related factors.

IR worsens during the development of type-2 diabetes mellitus (T2DM). This disease is a typical MetS, characterized by chronic hyperglycemia, impaired microcirculation, and the dysfunction of various organs and tissues, including muscle atrophy (8). Muscle atrophy seriously diminishes the quality of life in diabetic patients, whereas maintaining muscle homeostasis preserves overall body integrity and function (9). A study investigated the synergistic effect of NLRP3 inhibition combined with aerobic exercise and found that the intervention reduces pyroptosis, increases muscle mass, and improves strength and exercise performance. These findings provide a novel strategy for treating muscle attenuation in diabetes (10).

In addition to IR, age-induced obesity significantly increases the risk of T2DM and other MetS-related chronic diseases (11). By the end of 2023, people aged 60 and above reached 297 million, accounting for 21.1% of the population in China; in the United States, the population of the same age is projected to reach 88.5 million by 2050. The age-adjusted prevalence of obesity among U.S. adults was 42.4% in 2017–2018, with adults aged 40–59 having the highest prevalence of severe obesity (12) (13). T2DM, often overlapping with obesity, is one of the most prevalent aging-related diseases (14). Prophet models employed to forecast the prevalence of diabetes and obesity in 2030 using time-series data from the WHO Global Health Observatory identified three patterns of diabetes prevalence. Countries in cluster three were estimated to have the highest obesity (44.9%, 26.2-65.8%) and diabetes prevalence (25.3%, 18.3-32.6%) in 2030 (15). Therefore, the increasing rates of aging, obesity, and related chronic diseases highlight significant potential for exercise interventions in addressing these health issues.

Aging can also lead to muscle atrophy, which begins to occur in young and middle-aged individuals. Fortunately, exercise at an early age can increase lean body weight ratio and reduce body fat percentage. For example, resistance exercise improves body composition and alleviates age-related muscle atrophy in rats. The underlying mechanism may be related to the inhibition of pyroptosis in skeletal muscle and adipose tissues or the improvement of muscle protein metabolism (Fu et al.).

Notably, as a rapidly escalating global health concern, the prevalence of T2DM is projected to increase significantly in the near future. Jazieh et al. explored the intricate epigenetic modifications that contribute to the onset and exacerbation of T2DM and its complications, including diabetic retinopathy, atherosclerosis, neuropathy, and cardiomyopathy. These modifications influence key pathogenic processes such as obesity, insulin resistance, b-cell dysfunction, cellular senescence, and mitochondrial dysfunction. Importantly, lifestyle choices, such as exercise and diet, can promote and reduce the risk of T2DM through these epigenetic modifications. Li et al., provide a comprehensive elucidation of the molecular mechanisms underlying pyroptosis and the potential role of exercise in the treatment of T2DM and its complications through the modulation of anti-pyroptosis-associated inflammasome pathways.

Overall, various exercise methods, such as aerobic exercise (10), resistance exercise (Fu et al.), and other characteristic exercises (such as Tai Chi) (16), can effectively counteract pyroptosis, reduce inflammation, and prevent and improve MetS. Research on MetS highlights how anti-pyroptosis treatment influences exercise-induced metabolic health improvements, offering novel therapeutic targets for chronic diseases such as age-related obesity, muscle atrophy, and IR/T2DM. Thus, exercise presents promising strategies to inhibit pyroptosis, reduce inflammation, and enhance metabolic health.

## Author contributions

LG: Conceptualization, Investigation, Writing – original draft, Writing – review & editing. TT: Formal Analysis, Validation, Writing – review & editing. EZ: Supervision, Writing – review & editing.

## References

[1] Liu Y Cheng Y Xiang N Wang Z Li S Gong L. Aerobic exercise improves BKCa channel−mediated vasodilation in diabetic vascular smooth muscle via AMPK/Nrf2/HO−1 pathway. Acta Diabetologica. (2024) 61:425–34. doi: 10.1007/s00592-023-02210-z 38041787

[2] Zhou N Gong L Zhang E Wang X. Exploring exercise-driven exerkines: unraveling the regulation of metabolism and inflammation. PeerJ. (2024) 12:e17267. doi: 10.7717/peerj.17267 38699186 PMC11064867

[3] Huang Y Xu W Zhou R. NLRP3 inflammasome activation and cell death. Cell Mol Immunol. (2021) 18:2114–27. doi: 10.1038/s41423-021-00740-6 PMC8429580 34321623

[4] Rheinheimer J Souza B Cardoso NS Bauer AC Crispim D. Current role of the NLRP3 inflammasome on obesity and insulin resistance: A systematic review. Metabolism. (2017) 74:1–9. doi: 10.1016/j.metabol.2017.06.002 28764843

[5] Liu Z Gan L Xu Y Luo D Ren Q Wu S. Melatonin alleviates inflammasome-induced pyroptosis through inhibiting NF-κB/GSDMD signal in mice adipose tissue. J Pineal Res. (2017) 63(1):e12414. doi: 10.1111/jpi.12414 28398673

[6] Yang C-C Wu C-H Lin T-C Cheng Y-N Chang C-S Lee K-T. Inhibitory effect of PPARγ on NLRP3 inflammasome activation. Theranostics. (2021) 11:2424–41. doi: 10.7150/thno.46873 PMC7797672 33500734

[7] Romualdo M Nóbrega F Escrivão MAMS. Insulin resistance in obese children and adolescents. J Pediatr (Rio J). (2014) 90:600–7. doi: 10.1016/j.jped.2014.03.005 25019650

[8] Shen Y Li M Wang K Qi G Liu H Wang W. Diabetic muscular atrophy: Molecular mechanisms and promising therapies. Front Endocrinol (Lausanne). (2022) 13:917113. doi: 10.3389/fendo.2022.917113 35846289 PMC9279556

[9] Trierweiler H Kisielewicz G Jonasson TH Petterle RR Moreira CA Borba VZC. Sarcopenia: a chronic complication of type 2 diabetes mellitus. Diabetol Metab Syndr. (2018) 3:25. doi: 10.1186/s13098-018-0326-5 PMC5883537 29632617

[10] Yan X Fu P Zhang Y Ling D Reynolds L Hua W. MCC950 ameliorates diabetic muscle atrophy in mice by inhibition of pyroptosis and its synergistic effect with aerobic exercise. Molecules. (2024) 29:719. doi: 10.3390/molecules29030712 PMC10856337 38338456

[11] Umbayev B Saliev T Yantsen YS Yermekova A Olzhayev F Bulanin D. The role of cdc42 in the insulin and leptin pathways contributing to the development of age-related obesity. Nutrients. (2023) 15:4964. doi: 10.3390/nu15234964 38068822 PMC10707920

[12] Friedman AN Ogden CL Hales CM. Prevalence of obesity and CKD among adults in the United States, 2017-2020. Kidney Med. (2022) 5(1):100568. doi: 10.1016/j.xkme.2022.100568 PMC9881215 36713310

[13] Hales CM Carroll MD Fryar CD Ogden CL. Prevalence of obesity and severe obesity among adults: United States, 2017–2018. NCHS Data Brief. (2020) 2020(360):1–8. 32487284

[14] Zhao Y Yue R. Aging adipose tissue, insulin resistance, and type 2 diabetes. Biogerontology. (2024) 25:53–69. doi: 10.1007/s10522-023-10067-6 37725294

[15] Ampofo AG Boateng EB. Beyond 2020: Modelling obesity and diabetes prevalence. Diabetes Res Clin Pract. (2020) 167:108362. doi: 10.1016/j.diabres.2020.108362 32758618

[16] Hu S Hu Y Long P Li P Chen P Wang X. The effect of tai chi intervention on NLRP3 and its related antiviral inflammatory factors in the serum of patients with pre-diabetes. Front Immunol. (2022) 13:1026509. doi: 10.3389/fimmu.2022.1026509 36248820 PMC9554800

